# Insanity

**Published:** 1871-08

**Authors:** 


### Books Review.

*Insanity: Its Dependence on Physical Disease.* By John P. Gray, M. D.

This is a paper read before the Medical Society of the State of New York, at its Annual Meeting, February, 1871. The author first considers the causes of insanity, and shows by plainest reasoning and by abundant facts, that insanity has its origin in a large majority of instances at least in functional or organic change of the brain. He says, page 24, "The true and only method by which insanity can be studied is that followed in all other diseases. The physical lesions are the subjects of primary importance. These must be studied through physiology and pathology. The mental manifestations are here secondary and dependent. "Organs and tissues," says Dr. Gull, "have each their own life, and correlative with it, their own tendencies to disease, and their specific power and mode of repair," and "the purpose of our study is to trace these tendencies to their source on the one hand, and to their effects on the other."

"We say that insanity is a bodily disorder; that it is a disease of the brain. This does not imply that there is something to be thrown off, in the character of some morbid entity. It simply means that certain changes have taken place in the brain, or its investing membranes, which imply a departure from a healthy physiological action, and that in consequence of these changes there is more or less prolonged disturbance of the mind."

The paper is a very instructive one, and will do much towards correcting popular and professional error upon the subject of the causes and nature of insanity.

